# Evaluating the effect of polymer and titanium abutments on peri-implant tissues: A comparative clinical study

**DOI:** 10.6026/973206300211514

**Published:** 2025-06-30

**Authors:** Piyush Javiya, Chinju Punnen, Dipak Chaudhari, Paridhi Sharma, Snehamoy Pradhan, Deepak Sharma, Miral Mehta

**Affiliations:** 1 Department of Prosthodontics, Crown & Bridge, K. M. Shah Dental College & Hospital, Sumandeep Vidyapeeth Deemed to be University, Piparia, Waghodia, Vadodara, Gujarat, India; 2 Department of Oral and Maxillofacial Surgery, School of Dental Sciences, Krishna Vishwa Vidyapeeth (Deemed to be University), Taluka-Karad, Dist-Satara, Maharashtra, India; 3 Restorative and Cosmetic dentist, University of Pune, Smilebuilderz LLC, Lancaster, USA; 4 Department of Oral Medicine and Radiology, Triveni Institute of Dental Sciences Hospital and Research centre, Bilaspur, Chhattisgarh, India; 5 Intern, Kalinga Institute of Dental Sciences, KIIT Deemed to be University, Patia, Bhubaneswar, Odisha, 751024, India; 6 Periodontics, Graded Specialist, MDC Fatehgarh, Fatehgarh Military Station, Uttar Pradesh, India; 7 Department of Pediatric and Preventive Dentistry, Karnavati School of Dentistry, Karnavati University, Gandhinagar, Gujarat, India

**Keywords:** Polymer abutments, titanium abutments, peri-implant tissues, dental implants, soft tissue health

## Abstract

The evaluation between titanium and polymer abutments regarding peri-implant soft tissue health took place in a study involving 40 patients with posterior implants. Therefore, it is of interest to evaluate the plaque index (PI), Bleeding on probing (BOP) and Probing depth (PD) together with radiographic bone level assessments at three specified time points: baseline and 3 months as well as 6 months. The clinical assessment of Group B (polymer) at 6 months revealed superior outcomes including a decreased PI score to 0.88 alongside reduced BOP to 10% and contractions in PD up to 2.6 mm compared to 0.92 PI, 15% BOP and 2.9 mm PD. Group B participants showed smaller marginal bone loss than Group A with differences at 0.38 mm vs. 0.45 mm although results were not statistically meaningful. Patients obtained better results from polymer abutments although both types of abutment functioned properly.

## Background:

The durability of dental implants depends on osseointegration and healthy condition of tissues around implants. The abutment material functions as a vital determinant which shapes soft tissue reactions and hard tissue development surrounding implant fixture-prosthesis connections. Titanium abutments have gained extensive popularity because they display exceptional mechanical strength with excellent corrosion resistance and good biocompatibility [1, 2]. Patients with narrow oral tissue structures face aesthetic problems because titanium material generally creates colouring effects on the surrounding implant tissue areas [3]. The dental profession has incorporated high-performance polymers consisting of PEEK and multiple resin-based composites to address negative aesthetic aspects in dental constructions. These materials provide several benefits including optimal tissue compatibility as well as reduced plaque formation and better esthetic integration because they have natural tooth appearance and bone tissue similar elastic properties [4, 5]. The use of polymers in abutments leads to less inflammatory reactions in soft tissues than when using metal abutments [6]. The wide implementation of polymer abutments during clinical procedures is not accompanied by sufficient research that compares their tissue response to conventional titanium abutments. Therefore, it is of interest to assess how polymer abutments and titanium abutments affect peri-implant clinical assessments such as plaque accumulation and bleeding on probing, probing depth and marginal bone levels over six months.

## Materials and Methods:

This prospective clinical study was conducted on 40 patients requiring single posterior dental implants. Ethical clearance was obtained from the institutional review board and informed consent was taken from all participants before enrollment.

## Study design and grouping:

Patients were randomly divided into two equal groups (n = 20 each).

[1] Group A received implants restored with titanium abutments.

[2] Group B received implants restored with polymer abutments (*e.g.*, PEEK-based).

## Inclusion criteria:

[1] Patients aged between 25 and 55 years.

[2] Good general health and oral hygiene.

[3] Adequate bone volume to support standard-diameter implants.

[4] Non-smokers or light smokers (<10 cigarettes/day).

## Exclusion criteria:

[1] Systemic conditions affecting bone metabolism.

[2] History of periodontal disease in the implant site.

[3] Poor oral hygiene compliance.

[4] Pregnant or lactating women.

## Surgical procedure:

Standard two-stage implant placement was performed using a flap approach. All implants were placed by the same experienced surgeon to minimize procedural variability. A healing period of 3 months was allowed before prosthetic loading.

## Prosthetic phase:

After healing, either titanium or a polymer abutment was attached based on group allocation. The final crowns were cement-retained zirconia prostheses fabricated using CAD/CAM technology.

## Clinical evaluation:

Clinical parameters were assessed at baseline (prosthesis delivery), 3 months and 6 months post-loading.

[1] Plaque Index (PI)

[2] Bleeding on Probing (BOP)

[3] Probing Depth (PD)

[4] Marginal Bone Level (MBL) was measured using standardized digital periapical radiographs taken using the paralleling technique.

## Statistical analysis:

Data were analyzed using SPSS version 25.0. Descriptive statistics were calculated and intergroup comparisons were made using the independent t-test and chi-square test. A p-value of <0.05 was considered statistically significant.

## Results:

All 40 patients completed the study without complications. Both groups exhibited favorable peri-implant soft tissue responses throughout the observation period. Clinical parameters were recorded at baseline, 3 months and 6 months and are summarized below. At baseline, there were no statistically significant differences between the two groups in terms of plaque index (PI), bleeding on probing (BOP), probing depth (PD), or marginal bone loss (MBL). At 6 months, the polymer abutment group (Group B) demonstrated slightly better soft tissue outcomes compared to the titanium abutment group (Group A), though differences were not statistically significant. Plaque Index (PI) scores decreased over time in both groups, with Group B showing a marginally lower mean PI at 6 months (0.88 ± 0.24) compared to Group A (0.92 ± 0.21) (Table 1 - see PDF). Bleeding on probing (BOP) was observed in 15% of implants in Group A and 10% in Group B at the 6-month mark, indicating better tissue response in the polymer group (Table 1 - see PDF). Probing depth (PD) showed a slight reduction in both groups by the end of the study, with mean PD of 2.9 ± 0.4 mm in Group A and 2.6 ± 0.5 mm in Group B (Table 2 - see PDF). Marginal bone loss (MBL) measured radiographically was 0.45 ± 0.13 mm in the titanium group and 0.38 ± 0.10 mm in the polymer group at 6 months, showing a non-significant trend toward less bone resorption in the polymer abutment group (Table 2 - see PDF). These findings suggest a comparable clinical performance of both abutment materials, with slightly improved peri-implant tissue health in the polymer abutment group.

## Discussion:

The present study aimed to compare the effects of polymer and titanium abutments on peri-implant soft tissue health over a six-month period. Both materials exhibited satisfactory clinical outcomes; however, the polymer abutments demonstrated slightly better results in terms of soft tissue response and marginal bone preservation. Titanium has long been considered the gold standard for implant abutments due to its excellent mechanical strength, biocompatibility and corrosion resistance [1, 2]. Nevertheless, the esthetic limitations of titanium, particularly in patients with thin gingival biotypes, have prompted the development of alternative materials such as high-performance polymers [3, 4]. In our study, the polymer abutment group showed reduced bleeding on probing and probing depths, aligning with previous findings that suggest improved tissue adaptation around non-metallic abutments [5, 6]. Polymer abutments such as PEEK have a modulus of elasticity closer to that of natural bone, potentially reducing stress transmission to peri-implant tissues [7]. This biomechanical compatibility may contribute to the slightly lower marginal bone loss observed in the polymer group in our study. Similar outcomes were reported by Hürzeler *et al.* who found comparable osseointegration and tissue stability around PEEK abutments [8]. Plaque accumulation improved equally for both groups as time progressed though the scores were somewhat better in the polymer group. Some polymer materials possess smoother surfaces together with reduced biofilm affinity according to studies [9, 10]. Implant coatings are frequently applied to modulate tissue response and delivery of drugs. Copper (Cu)-containing coatings on dental implant abutments have been proposed to improve soft tissue integration and reduce the risk for peri-implant infections [11]. Some important drawbacks relating to polymer abutments need to be recognized even though their benefits are encouraging. The mechanical resilience of polymer abutments turns out to be sufficient for typical clinical applications yet slightly inferior to titanium strength in intense load areas [12]. The durability outcomes and wear resistance performance of polymer materials need additional research under both functional loading and long-term evaluation [13]. The findings from this research show that polymer abutments provide an effective substitute for titanium abutments especially in treatment situations requiring superior cosmetics or requiring attention of peri-implant tissues. Doctors must evaluate technical functionality with cosmetic requirements when deciding which abutment material to use. Additional studies must conduct prolonged observation periods and perform tissue sample pathology tests to determine the complete association between material structures and tissue components. The body of evidence would benefit from studies which implement larger sample sizes together with randomized controlled experimental designs according to [14, 15].

## Conclusion:

Peri-implant tissues generate suitable responses with abutments made from titanium as well as polymer materials. Polymer abutments performed better in regard to soft tissue healing and cosmetic results which indicates that they could serve as a titanium substitute in particular clinical applications for further consideration.
